# Traumatic brain injury induces an adaptive immune response in the meningeal transcriptome that is amplified by aging

**DOI:** 10.3389/fnins.2023.1210175

**Published:** 2023-07-31

**Authors:** Ruchelle G. Buenaventura, Alex C. Harvey, Mark P. Burns, Bevan S. Main

**Affiliations:** Laboratory for Brain Injury and Dementia, Department of Neuroscience, Georgetown University, Washington, DC, United States

**Keywords:** aging, traumatic brain injury (TBI), meninges, inflammation, immune response, adaptive immunity

## Abstract

Traumatic Brain Injury (TBI) is a major cause of disability and mortality, particularly among the elderly, yet our mechanistic understanding of how age renders the post-traumatic brain vulnerable to poor clinical outcomes and susceptible to neurological disease remains poorly understood. It is well established that dysregulated and sustained immune responses contribute to negative outcomes after TBI, however our understanding of the interactions between central and peripheral immune reservoirs is still unclear. The meninges serve as the interface between the brain and the immune system, facilitating important bi-directional roles in healthy and disease settings. It has been previously shown that disruption of this system exacerbates inflammation in age related neurodegenerative disorders such as Alzheimer’s disease, however we have an incomplete understanding of how the meningeal compartment influences immune responses after TBI. Here, we examine the meningeal tissue and its response to brain injury in young (3-months) and aged (18-months) mice. Utilizing a bioinformatic approach, high-throughput RNA sequencing demonstrates alterations in the meningeal transcriptome at sub-acute (7-days) and chronic (1 month) timepoints after injury. We find that age alone chronically exacerbates immunoglobulin production and B cell responses. After TBI, adaptive immune response genes are up-regulated in a temporal manner, with genes involved in T cell responses elevated sub-acutely, followed by increases in B cell related genes at chronic time points after injury. Pro-inflammatory cytokines are also implicated as contributing to the immune response in the meninges, with ingenuity pathway analysis identifying interferons as master regulators in aged mice compared to young mice following TBI. Collectively these data demonstrate the temporal series of meningeal specific signatures, providing insights into how age leads to worse neuroinflammatory outcomes in TBI.

## 1. Introduction

Traumatic Brain Injury (TBI) is a major cause of disability and mortality, posing a significant socioeconomic and public health burden, with an estimated 64–74 million people sustaining a TBI each year (Dewan et al., 2018). While the prevalence of TBI is centered around young adults and the elderly, the consequences of TBI are more severe in aged populations (Peterson et al., 2019). TBI in older adults is associated with higher morbidity and mortality (Testa et al., 2005; Thompson et al., 2006; McIntyre et al., 2013), slower recovery trajectories (Green et al., 2008), and worse functional, cognitive, and psychosocial outcomes post-injury (Susman et al., 2002; Papa et al., 2012; Gardner et al., 2018). These clinical findings highlight the need to understand of how age influences pathophysiological mechanisms in the post-traumatic brain.

Neuroinflammation is a key mechanism that contributes to cognitive dysfunction after TBI, with studies showing age-related exacerbation of inflammatory responses influence the severity of neurological deficits (Kumar et al., 2013; Erturk et al., 2016; Chou et al., 2018; Krukowski et al., 2018; Ritzel et al., 2019). Despite this knowledge of the detrimental effects of prolonged immune activation after TBI, our understanding of the interactions between central and peripheral immune reservoirs is still unclear. Recent studies have shown that the meninges, a tri-layered tissue between the brain and skull which physically envelops the central nervous system (CNS), is an active site for immunological processes. The meningeal tissue contains an array of immune cells, including macrophages, neutrophils, B lymphocytes and T lymphocytes [including CD4+, CD8+, γδ and regulatory T (Treg) subpopulations; O'Brien et al., 2017; Ribeiro et al., 2019]. All these immune cells have the capacity to traffic to the CNS parenchyma and generate robust inflammatory reactions in response to injury or disease (das Neves et al., 2021). Interestingly, disruption of meningeal vascular integrity and induction of a peripheral immune response is observed in mild TBI (Roth et al., 2014). Fluid-attenuated inversion recovery magnetic resonance imaging (FLAIR-MRI) shows focal enhancement of the meninges in 50% of patients with mild TBI (Roth et al., 2014; Russo et al., 2018). Temporally, this enhancement usually occurs in the early minutes and hours, before recovery within 1–3 weeks after injury (Roth et al., 2014; Russo et al., 2018; Turtzo et al., 2020). However, meningeal enhancement has been shown to persist in 17% of patients for several months (Russo et al., 2018), likely representing chronic inflammatory cascades occurring within this compartment after injury.

Alongside the capability to mediate inflammatory cascades, the meninges house lymphatic vessels that drain molecules and cells to peripheral lymph nodes (Louveau et al., 2015). Indeed, a recent study evidenced disruption of these meningeal lymphatics in an experimental model of mild TBI (Bolte et al., 2020). This was the first study to demonstrate that TBI-induced disruption of the meninges results in hyperactivation of innate CNS immune cells, and increased expression of proinflammatory cytokine related genes. Moreover, pre-existing meningeal lymphatic defects resulted in exacerbated TBI pathogenesis and more severe cognitive dysfunction (Bolte et al., 2020). Despite these studies, we currently have an incomplete understanding of the full array of acute and chronic meningeal derived responses. The meningeal lymphatic vessels can induce cytokine signaling from an array of immune cells which may influence social (Filiano et al., 2016), anxiety (Alves de Lima et al., 2020) and learning and memory behaviors (Ribeiro et al., 2019). The question of how the spectrum of TBI (mild/moderate/severe) shapes these cellular and molecular components in the meningeal environment over time, and whether or not this response is affected by aging, is still yet to be fully answered. Therefore, we investigated how the meningeal transcriptional environment is altered in the controlled cortical impact model of TBI. We utilized high-throughput RNA sequencing (RNAseq), and assessed sub-acute (7 days) and chronic (1 month) timepoints to gain insights on how meningeal gene expression signatures are altered by TBI and age. In addition, we sought to elucidate how chronic changes in the meningeal transcriptome may influence the potential for, or contribute to processes observed across the spectrum of known neurodegenerative disorders.

Here we report that the age alone is a significant factor in driving alterations in gene expression in the meningeal tissue. Specifically, aging influenced the adaptive immune response, upregulating genes involved in immunoglobulin production and the activation/regulation of B lymphocytes. In the context of TBI, both young and aged mice display sub-acute signatures of extracellular matrix remodeling, alongside upregulations in genes related to T lymphocyte responses at 7 days post-injury. This inflammatory signature is sustained at chronic timepoints, with B lymphocyte and interferon-based signatures observed at 1 month post TBI, a response that is unique to Aged TBI mice. Overall, these data demonstrate the time-course of meningeal specific signatures and provides insights into how age may lead to worse neuroinflammatory outcomes in TBI and how they may play a role in the context of other age-related neurodegenerative disorders.

## 2. Methods

### 2.1. Animals

Wildtype C57BL/6 male mice were purchased from Jackson Laboratories (Bar Harbor, ME) and were 3–4 months or 18–19 months of age at the time of injury. Mice were housed 5 per cage under 12:12 h light:dark cycles with food and water available *ad libitum*, and maintained at a temperature of 22°C–24°C and 40%–60% humidity. All procedures were conducted in accordance with protocols approved by the Georgetown University Animal Care and Use Committee. Experiments adhered to guidelines from the Guide for the Care and Use of Laboratory Animals, U.S. Department of Health and Human Services.

### 2.2. Controlled cortical impact mouse model of TBI

Controlled cortical impact (CCI) was conducted as previously described (Main et al., 2018; Korthas et al., 2022). Briefly, surgical anesthesia was induced using 4% isoflurane with maintenance at 2%, at flow rate of 1–1.5 L/min in freely breathing oxygen. The anesthetized animal was mounted in a stereotaxic frame with built-in heating bed to maintain body temperature at 37°C for the duration of the surgery. The surgical site over the top of the head was clipped and aseptically sterilized with alcohol swabs and topical iodine application. A 10 mm midline incision was made over the skull, the skin and fascia reflected and craniotomy performed (4 mm) on the central aspect of the left parietal bone. The impounder tip of a Leica StereoOne Impactor was sterilized, positioned to the surface of the exposed dura, and set to impact the cortical surface at 5.25-m/s velocity, 1.5 mm tissue deformation. Sham animals received isoflurane anesthesia, skin incision, and reflection, but no craniotomy or impact. After injury, the incision was closed with wound clips, anesthesia discontinued, 1 mL saline administered by intraperitoneal (i.p) injection and mouse placed in a heated cage to maintain normothermia for a 45 min recovery period. All animals were monitored carefully for 4 h after surgery and then daily.

### 2.3. Experimental design

**Figure 1:**
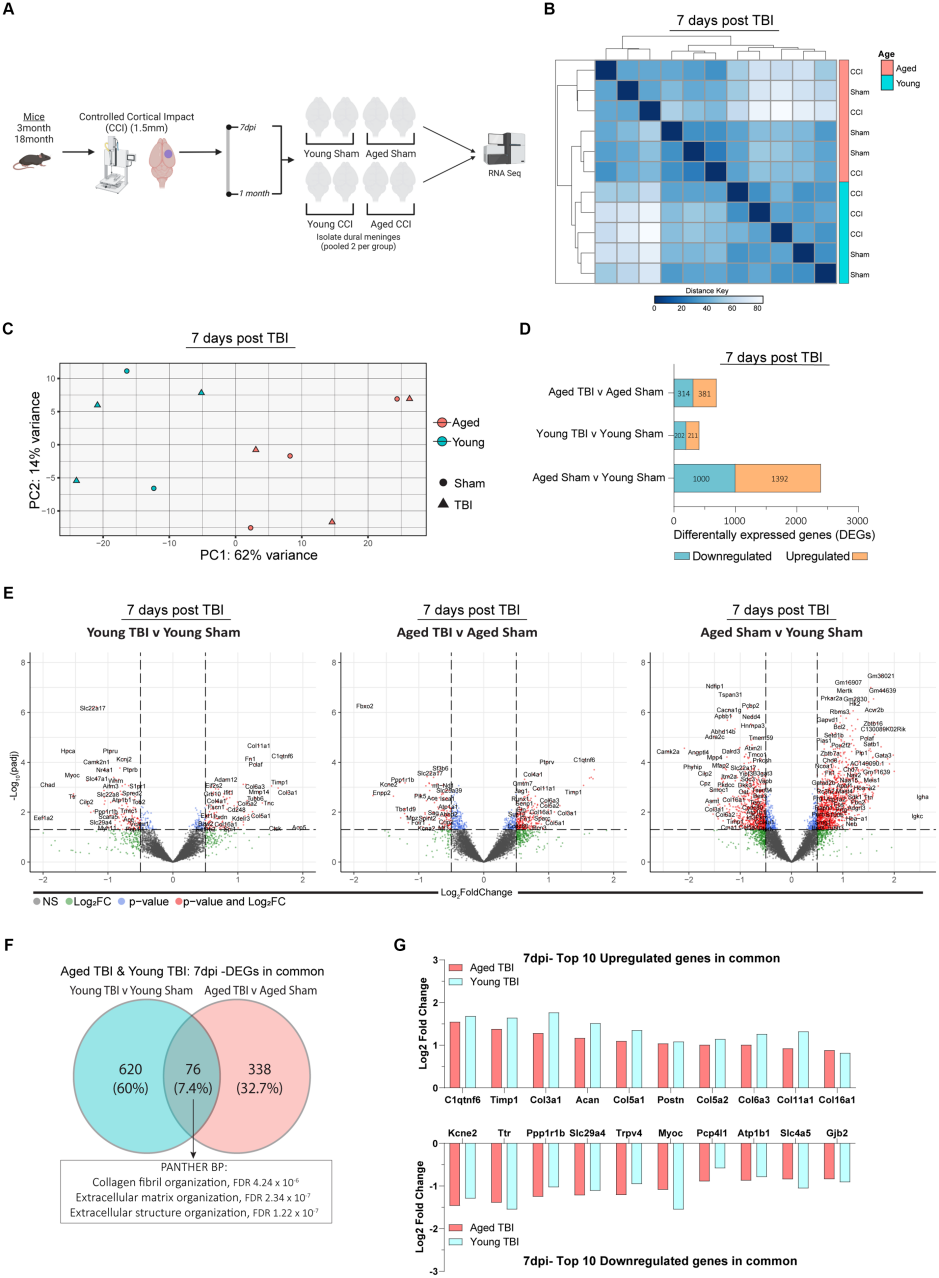
Sub-acute effects of TBI and aging on the meningeal transcriptome. **(A)** Schematic of the experimental design. Young (3 m) and Aged (18 m) male mice were subject to sham or TBI before dural meninges were collected at 7 days post-injury and analyzed by RNA-seq. **(B)** Hierarchical clustering using Euclidean distance reveals separation at 7 days post-injury. **(C)** Principal component analysis (PCA) at 7 days post-injury revels greatest separation is observed as result of age, followed by injury at 7 days post-injury. **(D)** Bar plot depicting significantly downregulated and upregulated genes for each FIGURE 1 (Continued)comparison at 7 days post injury. **(E)** Volcano plots show the spread of these significantly differentially expressed genes between each group comparison at 7 days post injury. FDR and *p*-values were calculated with DESeq2 using the Wald test for significance following fitting to a negative binomial linear model and the Benjamini-Hochberg procedure to control for false discoveries. **(F)** Venn diagram demonstrating overlap of DEGs in Young and Aged TBI mice at 7 days post injury. **(G)** Log2 fold change of the top 10 upregulated and downregulated genes that both Young and Aged mice have in common after TBI.

In this study design, four experimental groups; Young Sham, Aged Sham, Young TBI and Aged TBI were assessed across two different time points of 7 days and 1 month post TBI. Experimentally, TBI or sham surgery was performed on *n* = 6 mice for each time points. Due to RNA yield from each tissue, dural meninges from 2 mice were combined to create 1 biological replicate at each time point (Figure 1A).

### 2.4. Collection of dural meningeal tissue

Mice were euthanized with CO2, followed by bilateral thoracotomy and transcardiac perfusion with ice-cold 1× PBS, using a peristaltic pump set to a flow rate of 5 mL/min. Following perfusion, skin and muscle were stripped from the outer skull. Skullcaps were removed via the ventral side of the jaw, with the lower jaw removed from the mandibular junction, before incisions dorsal to the ear canal, removal of the brain and collection of the skullcap containing the adherent meninges, as previously described (Louveau et al., 2018a). Skullcaps were immediately placed in a 10-mm petri dish with 1× PBS, and using a stereomicroscope (M80, Leica), meninges were scraped and removed intact (dura mater and arachnoid) using Dumont #5 forceps (Fine Science Tools). After meningeal collection from the skullcap, meninges were placed in 500 μL TRIzol Reagent (15596018, Life Technologies), immediately snap-frozen and stored at −80°C until further use.

### 2.5. RNA extraction

For each of the four experimental groups (Young Sham, Aged Sham, Young TBI and Aged TBI), two meningeal samples were pooled to create one biological replicate. RNA was then extracted from each meningeal biological replicate using TRIzol® reagent (15596026, Invitrogen), as described in detail previously (Buenaventura et al., 2022). Concentration and purity of the RNA was assessed using a NanoDrop 1,000 spectrophotometer (ThermoScientific). RNA was aliquoted stored immediately at −80°C before use in RT-QPCR applications and high throughput RNA sequencing pipelines (Sloley et al., 2021).

### 2.6. RNA-seq analysis

The raw RNA-Seq reads (FASTQ files) were aligned to the mouse reference genome (mm10) using STAR (Dobin et al., 2013) and expression counts per transcript quantified using eXpress (Roberts and Pachter, 2013). From the raw counts per transcript, we used DESeq2 (Love et al., 2014) to normalize data and estimate differential expression between groups, and generate datasets containing contrasts. Briefly, from raw counts our DESeq2 pipeline estimated size factors (to account for differences in library depth) and gene-wise dispersions before shrinking these estimates to generate an accurate dispersion to model the counts. Then we fit the negative binomial model for differential expression testing to generate contrasts per region and log2 fold changes (LFC) for each sample group, before performing hypothesis testing using the Wald test. To improve power detection, outliers of genes whose mean of normalized counts was below the default threshold were removed using Cook’s distance cutoffs. To determine significance, the Wald test takes the LFC and divides it by its standard error, resulting in a z-statistic. This z-statistic is then compared to a standard normal distribution, in order to compute a *p*-value for each specific gene. To correct for multiple testing and control the false discovery rate (FDR), the Benjamini-Hochberg (BH) algorithm was used set to a threshold of 0.1. Results tables were generated for pairwise comparisons of interest.

Following DESeq2 analysis and generation of variance stabilized contrasts with statistically significant log2 fold changes, principal component analysis (PCA) and volcano plots and were generated from datasets using ggplot2, lattice and enhanced volcano R packages. Heatmaps were generated using the pheatmap R package, and Morpheus software.^1^

The Gene Ontology (GO) Resource^2^ was used for analysis of ontologies, biological functions and relationships (Ashburner et al., 2000; Gene Ontology, 2021). Enrichment analysis of genes was performed using the PANTHER (Protein ANalysis THrough Evolutionary Relationships) classification system (Mi et al., 2019). Using PANTHER (version 17.0), gene sets were uploaded for biological process (BP) analysis to identify hierarchical relations between over-represented or enriched functional classes. Fisher’s exact test followed by FDR correction determined statistically enriched categories. Dot plots were used to visualized this analysis, using ggplot2 R package.

CIBERSORTx was used to deconvolve meningeal bulk RNA-Seq data *in silico* against validated mouse (Mostafavi et al., 2016, Immgen GeoAccession: GSE75202) and human (Newman et al., 2015). reference profiles, which distinguish distinct immune cell subpopulations (Chen et al., 2018). Using CIBERSORTx impute cell fractions tool, bulk RNA-Seq data were inputted in CPM (counts per million) format and batch corrected under B-mode against the validated microarray matrix to reduce the impact of cross-platform variation (Newman et al., 2015; Steen et al., 2020). Bulk RNA-Seq data were averaged per sample phenotype, deconvolved separately against mouse and human immune cell populations, and presented in relative percentage format for each existing cell expression fraction.

Ingenuity Pathways Analysis (IPA, Qiagen), was performed to identify significant upstream regulators and canonical pathways (Kramer et al., 2014). Across our comparison groups of TBI and age, significantly expressed gene names and fold changes were submitted to compare expression patterns in our dataset to the IPA database. IPA results for canonical pathways and master regulators with *p* < 0.05 were considered significant.

The GWAS Catalog^3^ procured by the European Bioinformatic Institute and the National Human Genome Research Institute (Sollis et al., 2023), was used to identify genes associated with diseases (Neurodegenerative, Alzheimer’s, Parkinson’s) or molecular traits (inflammatory biomarker, memory, cognition). We then group wise compared significantly DEGs in our datasets with GWAS identified genes to assess translation of our results to known neurodegenerative processes. Visualization of these overlaps was performed using circus plots made with the R packages circlize and dichromat.

### 2.7. Statistics

Statistical testing for RNAseq experiments is discussed in the relevant methods section. All sequencing analysis was performed using RStudio (v4.1.3) and GraphPad Prism (v9.5.0). The criteria for statistical significance were preset at *p* < 0.05 for analysis including IPA and gene ontology enrichment.

## 3. Results

### 3.1. The subacute effects of TBI and aging on the meningeal transcriptome

It is well established that age and aging related processes remain key factors that influence outcomes after TBI. Along with age, neuro-immune cascades from both central and peripheral mediators influence functional recovery timeframes. Importantly, these responses are time and context dependent, therefore we sought to investigate the temporal series of meningeal transcriptomic responses, and how age and post-injury interval effect these responses. Young and aged C57BL/6 wildtype male mice were subject to TBI, before assessment of the meningeal transcriptional response at 7 days post-injury. Hierarchical clustering of the aligned and normalized read counts indicated that age was the main factor driving separation of differential gene expression (Figure 1B). This was confirmed with principal component analysis (PCA) showing young and aged groups clustered furthest apart. While age was a clear separator, mice that received TBI were also spread, with Young TBI and Aged TBI mice having varied clusters at locations separated clear of each other (Figure 1C). Indeed, when we looked at the number of differentially expressed genes (DEGs) between experimental comparison groups, Young TBI mice have only 413 DEGs (211 upregulated, 202 downregulated), while Aged TBI mice display 695 DEGs (381 upregulated, 314 downregulated) at 7 days post-injury (Figure 1D; Supplementary Table S1). Interestingly, 2,392 differentially expressed genes (1,392 upregulated, 1,000 downregulated) were observed when comparing Aged Sham mice to Young Sham mice (Figure 1D; Supplementary Table S1). This effect was further confirmed via volcano plot visualization, showing that not only is there more significantly altered genes (red), but the overall level of transcriptomic variation is higher as a result of aging (Figure 1E). To gain an understanding of the type of immune cells affected by age and TBI, we deconvolved bulk RNAseq data using *in silico* CIBERSORTx software. At 7 days post-injury, meningeal macrophage responses are enhanced by TBI in young mice, a response which is dampened in aged TBI mice (Supplementary Figure 1; Supplementary Table S1). Comparison of DEGs in Young TBI and Aged TBI groups revealed 76 overlapping genes (Figure 1F). PANTHER ontology analysis showed these genes are involved in extracellular matrix (GO:0030198) and collagen organization (GO:0030199) biological processes, with significant fold enrichment (F.E 13.8–17.8) in these categories (Figure 1F; Supplementary Table S7). The extracellular matrix is further characterized by a trend of decreased subacute laminin expression, including lama, lamb, and lamc genes independent of age (Supplementary Table S7). Of the 76 overlapping genes, the top upregulated genes based on Log2 fold change are collagen related genes in Young TBI and Aged TBI (Figure 1G), further evidencing the potential extracellular matrix repair and resolution in the meninges early after injury.

### 3.2. Meningeal upstream regulators and canonical pathways in sub-acute TBI

**Figure 2:**
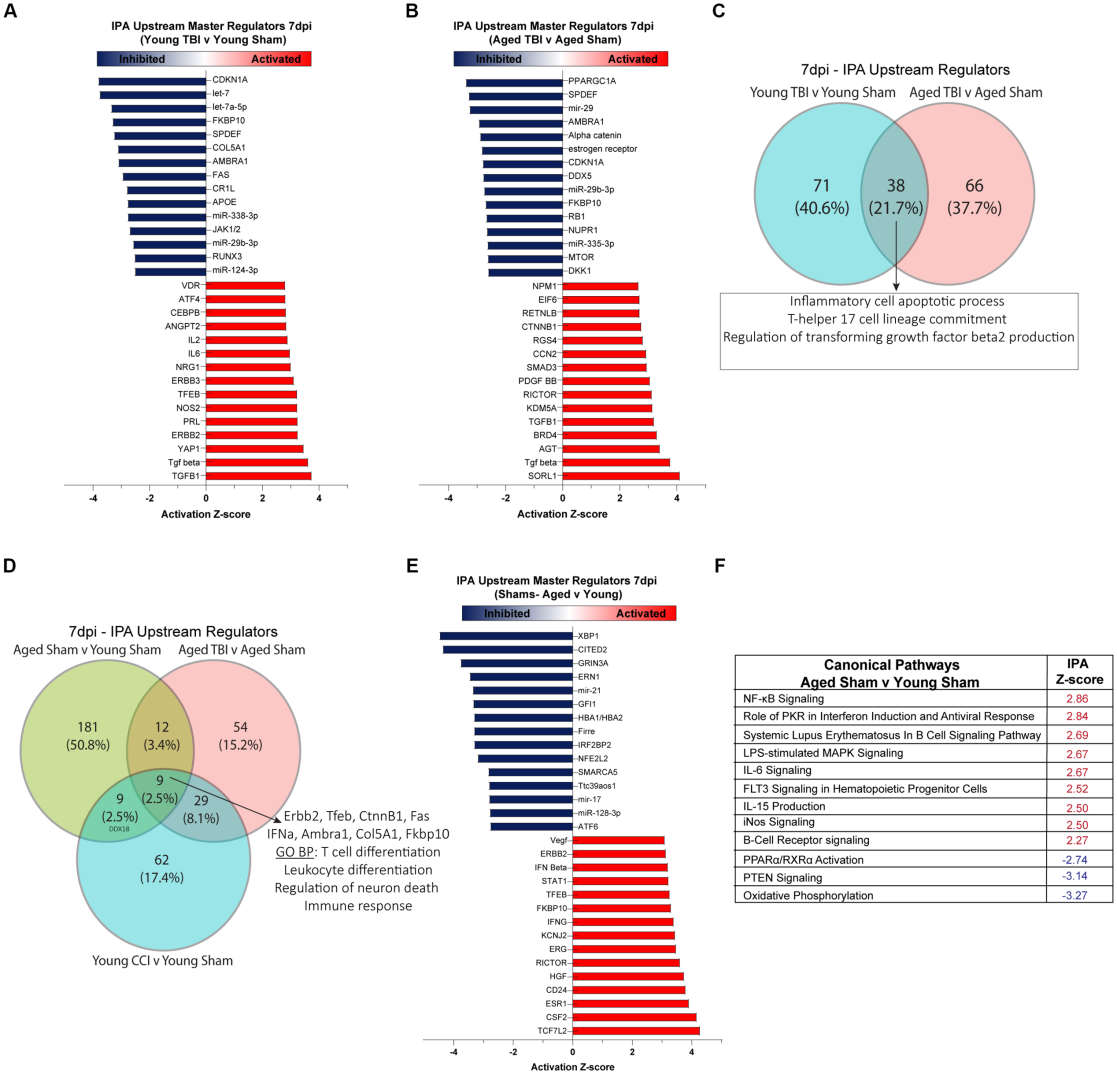
Meningeal upstream regulators and canonical pathways in sub-acute TBI and aging. Ingenuity pathway analysis (IPA) of upstream regulators in **(A)** Young TBI v Young Sham and **(B)** Aged TBI v Aged Sham groups. Red indicates a significantly elevated z-score signifying that the regulator is activated by TBI. Blue indicates significantly repressed z-score signifying that the regulator is inhibited by TBI. Top 15 activated and inhibited regulators shown for each group. **(C)** Venn diagram demonstrating overlap of upstream regulators 7 days post injury. TBI in Young and Aged mice share 38 conserved regulators, with gene ontology (GO) analysis showing these regulators have biological processes that apoptosis and T cell responses. **(D)** Venn diagram demonstrating overlap of upstream regulators in all TBI and Aging groups at 7 days post injury. Across all groups of aging alone and TBI, 9 conserved regulators are shared, with biological processes that include regulation of neuronal death and leukocyte and T cell immune responses. **(E)** Top 15 upstream regulators that are activated (red) or inhibited (blue) by aging. **(F)** Z-score of significant canonical pathways that are activated (red) or inhibited (blue) as a function of aging.

To gain insights into mechanisms and pathways associated with the DEGs in our datasets, we performed Ingenuity Pathway Analysis (IPA) on young and aged comparison groups at 7 days post TBI. All raw outputs from IPA are included in Supplementary Table S2. IPA showed 71 unique genetic regulators modulated in Young TBI mice. Assessment of significantly activated upstream regulators (*p* < 0.05; absolute Z-Score > 2) showed lymphocyte and acute immune signatures in Young TBI mice, evidenced by IL2, IL5, CEBPB and ANGPT2 activation (Figure 2A). Enhancing the notion of upstream immune responses, cytokines (TGFB) and oxidative stress mediators (NOS2, AT4) were also activated in Young TBI mice. Alongside activation, a host of upstream mediators were inhibited (*p* < 0.05; absolute Z-Score > −2), including complement receptor *Cr11* and *miR-338-3p, miR-29b-3p, miR-124-3p* which inhibit MAPK, JAK/STAT and NF-kB signaling (Figure 2A; Supplementary Table S8). At 7 days post injury, Aged TBI mice have 66 unique upstream regulators compared to Aged Sham mice. Aged TBI also have significant activation of upstream cytokines including TGFB, as well as the endosomal trafficking and risk factor for Alzheimer’s disease gene SORL1 (Figure 2B). The glucose metabolism gene PPARGC1A had the lowest Z-score, demonstrating inhibition upstream, followed by the inhibition of alpha catenin family which plays a key role in stabilizing adherens junctions and sealing membranes in the maintenance of Blood Brain Barrier (BBB) integrity (Figure 2B). Comparison upstream regulators in both Young and Aged TBI groups showed 38 (21.7%) shared regulators, with these mediators having ontology profiles that evidence their role in apoptotic processes (GO:0006925) and T lymphocyte driven immune processes (GO:0072540; Figure 2C; Supplementary Table S2). As aging profoundly affected meningeal gene expression (Figure 1), we also assessed the influence of age on upstream regulators and canonical pathways. Notably, age alone had 181 unique upstream regulators, which accounts for over 50% of the total regulators when all three groups are compared (Figure 2D). Key upstream master regulators that are activated due to aging include type-1 and type-2 interferons (IFNG; Supplementary Table S8), regulators of NF-kB signaling (HGF) and T cells (ESR1, CSF2), as well as transcriptional regulators of cell proliferation cascades (ERG, ERBB2, RICTOR, CD23, TCF7L2; Figure 2E). Similarly, when assessing significant canonical pathways modulated by aging, amplified inflammatory cascades are observed, including NF-kB signaling, PKR interferon induction, B cell and iNOS signaling (Figure 2F). In addition, oxidative phosphorylation, PTEN signaling, and PPARα/RXRα pathways were inhibited canonical pathway, indicating depleted metabolic regulation in aged mice (Figure 2F).

### 3.3. Chronic effects of TBI on the meningeal transcriptome

**Figure 3:**
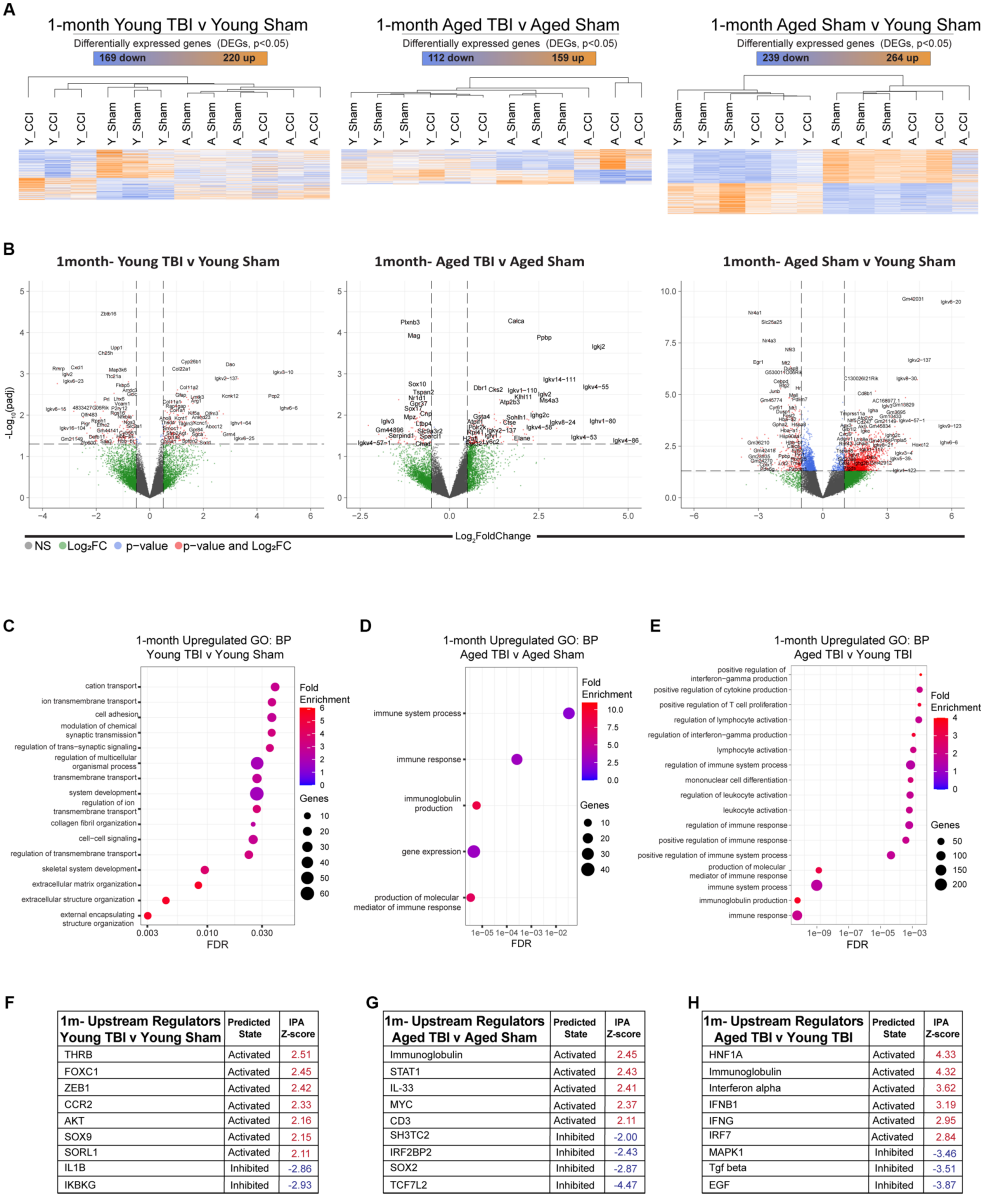
Chronic effects of TBI on the meningeal transcriptome. Young (3 m) and Aged (18 m) male mice were subject to sham or TBI before dural meninges were collected at 1 month post injury and analyzed by RNA-seq. **(A)** Heatmap with all significantly differentially expressed genes (DEGs) across injury and age groups. Color scale bar values represent Z-value (normalized standard deviations from the mean) for expression relative to the overall mean expression. Numbers of significantly upregulated and downregulated genes are indicated inside color scale bar. **(B)** Volcano plots show the spread of these significantly differentially expressed genes. **(C)** Dot plot of gene sets obtained by functional enrichment of significantly altered genes in each group as a factor of TBI. Using gene ontology (GO) analysis of biological processes (BP), aged TBI shows upregulations in immune processes and immunoglobulin production. **(D)** Young TBI has highly enriched matrix and structural organization processes. **(E)** Comparisons of both injury and age, shows aged TBI responses have increased immune responses including interferon and immunoglobin production and increased leukocyte responses. Node size corresponds to the number of genes in each ontology term. Color of each dot is the gene ratio compared to the total gene number. **(F–H)** IPA and z-score of top upstream regulators that are activated (red) or inhibited (blue) as a function TBI and aging.

Young and aged CB57BL/6 wildtype male mice were subject to TBI, before assessment of the meningeal transcriptional response at 1 month post injury (Supplementary Table S3). Heatmap clustering of significantly DEGs at showed similar levels of genetic expression in both Aged TBI (112 upregulated, 159 downregulated) and Young TBI (220 upregulated, 169 downregulated) groups. Similar to 7dpi, the most amount of DEGs was observed as a factor of aging alone (264 upregulated, 239 downregulated; Figure 3A; Supplementary Table S3). This was further evidenced by volcano plot analysis, which also showed that the highest Log2 FC genes in all groups belonged to antibody production, with *Ighv6-6, Igkv4-86*, and *Igkv6-20* the highest significantly upregulated gene in Young TBI, Aged TBI, and Aged Sham groups, respectively (Figure 3B). *In silico* deconvolution using CIBERSORTx, showed that at this chronic timepoint, young TBI mice still contain elevated macrophage contributions compared to Aged TBI mice. It also revealed a unique contribution of monocytes and plasma cells in Aged TBI compared to Young TBI (Supplementary Figure 1; Supplementary Table S3). Assessment of gene ontology (GO) biological processes (BP) showed that Young TBI mice display elevated activation of extracellular matrix processes, with matrix structure (GO:0030198) and collagen fibril organization (GO:0030199) significantly enriched (Figure 3C; Supplementary Table S7). Aged TBI mice display enhanced inflammatory based responses with immune response, immunoglobulin production (GO:0002377) and production of molecular mediators in immune responses (GO:0002440) significantly enriched (Figure 3D). This immune phenotype after TBI may be specific to Aged TBI mice, with assessment of Aged TBI v Young TBI revealing that the Aged TBI differentiates through enriched immune responses in cytokine production (GO:0006955), T cell/lymphocyte proliferation (GO:0042102), interferon-gamma (GO:0032729) and immunoglobulin production (GO:0002377; Figure 3E; Supplementary Tables S5, S8). Ingenuity Pathway Analysis (IPA) of upstream regulators (Supplementary Table S4) in Young TBI mice at 1 month post injury showed activation of mediators involved in immune suppression (FOXC1), cytokine expression [ZEB1, AKT, SOX9, SORL1, and monocyte recruitment (CCR2)], while inhibition of mediators (IL1B, IKBKG) involved in pro-inflammatory responses (Figure 3F). Meanwhile, Aged TBI mice display activated upstream regulators including immunoglobulins, cytokines (IL33) and T cell receptors (CD3), with inhibited regulators including IRF2BP2, SOX2, and WNT pathway transcription factor (TCF7L2) at 1 month post injury (Figure 3G). This enhanced inflammatory response is confirmed when assessing upstream regulators that are unique to Aged TBI vs. Young TBI. Specifically, a robust interferon master regulator signature is observed upstream, with activation in the interferon alpha family, IFNB1 (interferon beta), IFNG (interferon gamma) and IRF7 (interferon regulatory factor 7; Figure 3H; Supplementary Table S8). In contrast ani-inflammatory mediators including (TFGB, EGF) are uniquely inhibited in Aged TBI mice 1 month post injury (Figure 3H).

### 3.4. The impact of age on the meningeal transcriptome

**Figure 4:**
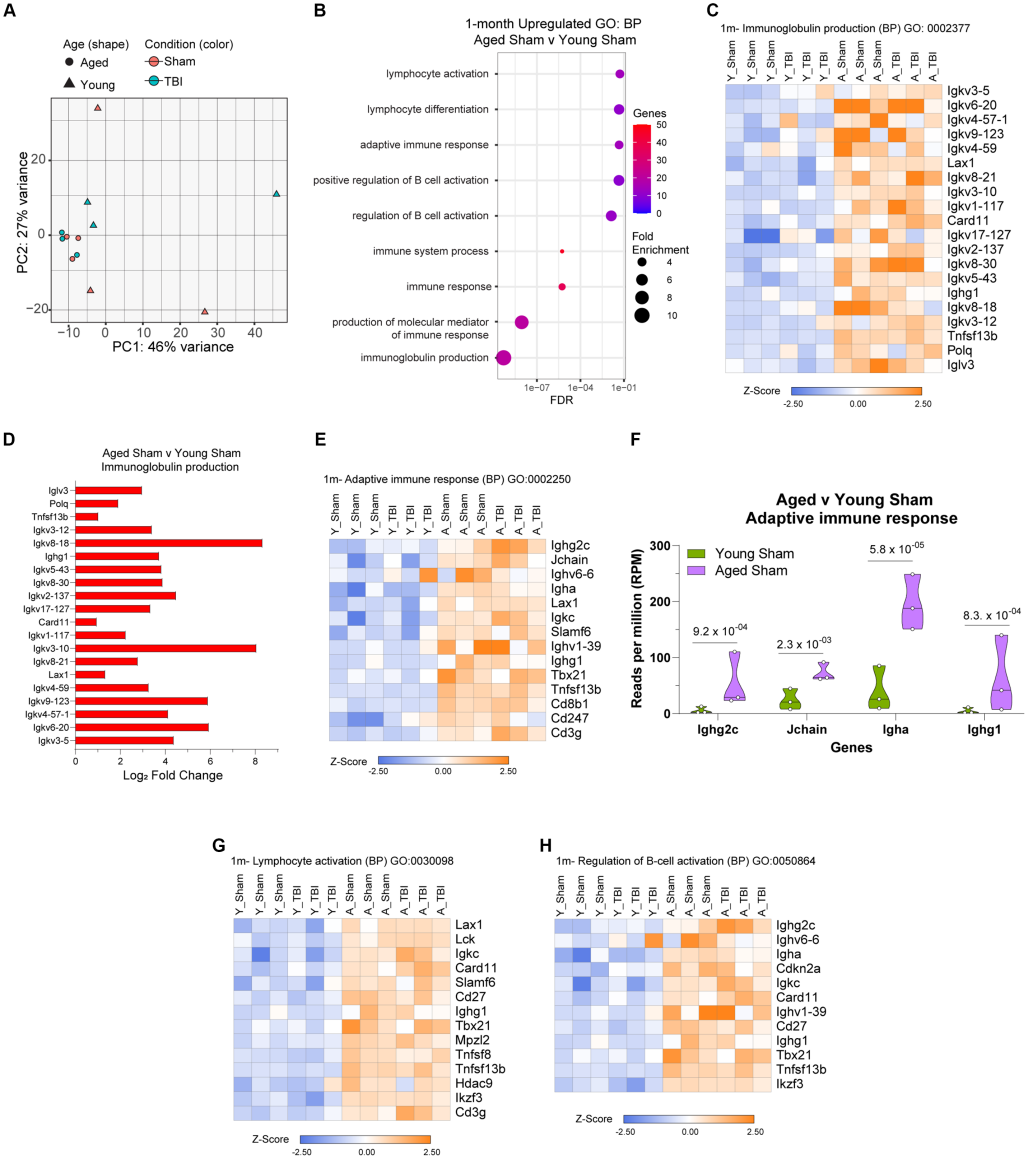
The influence of aging on the meningeal transcriptome. **(A)** Principle component analysis (PCA) revels greatest separation is observed as result of age at 1 month post injury. **(B)** Dot plot of gene sets obtained by functional enrichment of significantly altered genes in aged mice compared to young mice. Node size corresponds to the number of genes in each ontology term. Color of each dot is the gene ratio compared to the total gene number. **(C)** Heat map displaying the top differentiated genes in immunoglobulin production biological process (BP). **(D)** Bar graph demonstrating the Log2 Fold Change of genes involved in immunoglobulin production in aged mice compared to young mice. **(E)** Heat map displaying the top differentiated genes in adaptive immune response biological process (BP). **(F)** Violin plot depicting counts of significantly increased adaptive immune response in response to age. The number above each comparison on the graph represents the adjusted value of p calculated for each gene using DESeq2. The central line within each plot represents the median of the data set. Heat map displaying the top differentiated genes in **(G)** lymphocyte activation and **(H)** regulation of B cell activation biological process (BP). For all heatmaps, color scale bar values represent Z-value (normalized standard deviations from the mean) for expression relative to the overall mean expression.

Given aging itself resulted in substantially different gene expression patterns (Figure 4A), we decided to look more closely at these differences within the bulk RNA-seq dataset. To gain insights into the molecular pathways associated with age related changes in gene expression, we performed gene ontology (GO) enrichment. Analysis of biological process (BP) categories revealed that age alone resulted in elevated inflammatory signatures, including immunoglobulin production (GO:0002377), immune system process (GO:0002376) and regulation of B cell activation (GO:0050864; Figure 4B; Supplementary Table S5), compared to Young Sham mice. The genetic components related to each of these processes were further profiled and show a significant pattern of separation in overall adaptive immune upregulation in aged mice (Figures 4C–H). Key immunoglobulin clusters are highly active (Figure 4C), with regulators like *Igkv8-18* and *Igkv3-10* presenting 8-fold upregulation (Figure 4D). The constant secretion of antigen processes indicates that the meninges in aged mice have a baseline adaptive immunity that may be constantly activated for defense. Adaptive immune responses are also populated by T cell mediators (*Cd8b1, Cd247, Cd3g*), leukocytes (*Lax1*), and immunoglobulin production (*Ighg2c, Igha, Ighv6-6*; Figure 4E). It is evident that the genetic expression of prominent adaptive immune response activity by *Ighg2c, Jchain, Igha*, and *Ighg1* in aged mice is significantly upregulated compared to young mice (Figure 4F). Aged lymphocyte activation is characterized by increased expression of *Lax1, Lck, Igkc, Card11, Slamf6, Cd27, Ighg1, Tbx21, Mpzl2, Tnfsf8, Hdac9, Ikzf3, Cd3g* (Figure 4G) and aged B cell activation is regulated by an increase of *Ighg2c, Ughv6-6, Igha, Cdkn2a, Ugkc, Card11, Ighv1-39, Cd27, Tbx21, Tnsfs13b,* and *Ikzf3* (Figure 4H). These transcriptomic profiles validate the chronic upregulation of the adaptive immune response and provide context for the immunological status of the meninges in aging.

### 3.5. Meningeal architecture of TBI is associated with alterations to neurobiological traits

**Figure 5:**
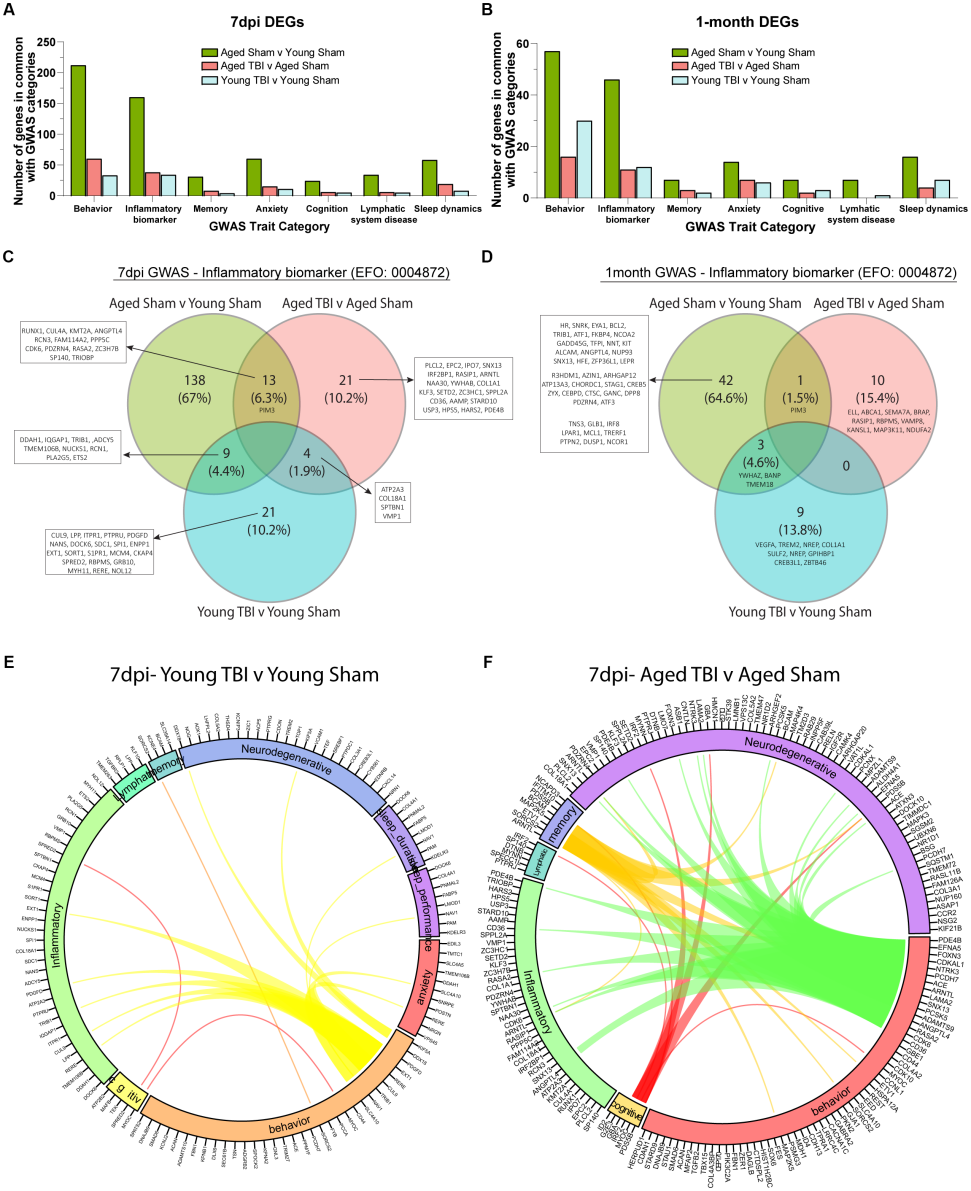
Meningeal architecture of TBI is associated with alterations to neurobiological traits. **(A)** Bar graph showing the number of differentially expressed genes at 7 days post injury that overlap with known trait categories observed in genome wide association (GWAS) studies. **(B)** Bar graph showing the number of differentially expressed genes at 1 month post injury that overlap with known trait categories observed in GWAS studies. **(C,D)** Venn diagram demonstrating the number of genes at 7 days and 1 month post injury that have been identified in GWAS studies of inflammatory biomarkers. **(E,F)** Circos plot depicting how overlapping differentially expressed genes at 7dpi (in Young TBI and Aged TBI) are associated with inflammatory and cognitive traits as identified in GWAS studies. The proportion of the circle’s circumference allocated to each trait represents the number of genes associated with that trait that are also differentially expressed in both aged and TBI mice. The lines connecting genes within the circle indicate which genes were shared among trait signatures.

Transcriptomic analyses between age and TBI at sub-acute and chronic time points reveal upregulated inflammatory activity in the meninges. To further understand how the inflammatory signature in aging and TBI is related to functional consequences, we evaluated meningeal genomic data through a human pathophysiology context. Genome-wide association study (GWAS) analysis of common genes with high activity in the aging brain show significant overlap in a number of functional traits including behavior, neurodegeneration, inflammation, cognition, memory, anxiety, and lymphatic system disease (Figures 5A,B; Supplementary Table S6). To further investigate the inflammatory biomarkers from TBI, we compared the transcriptomic profiles of each time point and assessed for similarities. The aged brain has a unique inflammatory profile at both time points (67% at 7dpi and 64.7% at 1mpi), which provides evidence of baseline inflammation with aging independently (Figures 5C,D; Supplementary Table S6). The differential expression of inflammatory biomarkers in TBI with age also show variant profiles at the subacute (10.2% in Young TBI and 10.2% in Aged TBI) and chronic (13.8% in Young TBI and 15.4% in Aged TBI) time points (Figures 5C,D). Acutely, we found 13 genes in common between Aged Sham and Aged TBI mice (*RUNX1, CUL4A, KMT2A, ANGPTL4, RCN3, FAM114A2, PPP5C, CDK6, PDZRN4, RASA2, ZC3H7B, SP140, TRIOBP*), 9 genes in common between Aged Sham and Young TBI mice (*DDAH1, IQGAP1, TRIB1, ADCY5, TMEM106B, NUCKS1, RCN1, PLA2G5, ETS2*), and 4 genes in common between both TBI groups (*ATP2A3, COL18A1, SPTBN1, VMP1*; Figure 5C). Chronically, we found 1 common gene between Aged Sham and Aged TBI mice (*PIM3*) and 3 similar genes between Aged Sham and Young TBI mice (*YWHAZ, BANP, TMEM18*; Figure 5D). These transcriptomic differences reveal unique inflammatory responses with age and TBI evidenced by specific genomic trait profiles per condition. We then assessed the differential trait interaction patterns with age as a factor of TBI (Figures 5E,F; Supplementary Table S6). Compared to Young TBI (Figure 5E), Aged TBI (Figure 5F) appears to have increased dynamic connectivity in relation to behavioral genomics. Acutely, Young TBI reveal behavior-related genes *SLC4A10, NAV1, TRIB1, CUL9, RERE, EXT1, PDGFD, DDX18, KIF5A* are linked to inflammation, neurodegeneration, sleep duration, sleep performance, and anxiety (Figure 5E). Cognitive disability is also related to behavior and inflammation through *SPRED2* and *MYOC*. In comparison, Aged TBI have more prominent genomic associated overlaps, where behavior-related genes *CD36, CDK6, RASA2, ANGPTL4, PCSK5, SNX13, LAMA2, ARNTL, ACE, PCDH7, NTRK3, CDKAL1, FOXN3, EFNA,* and *PDE4B* are linked to inflammation and neurodegeneration. Memory deficits are related to altered behavior, inflammation, and neurodegeneration through *ARNTL, SORCS2, ETV1, MAP2K5, BCAM, PSD5B, IFITIM10,* and *NCAPD3,* while cognitive disability is linked to neurodegeneration, behavior, and lymphatics through *ID2, G2E3, GBA, IRF2, MYOC,* and *PSD5B* (Figure 5F). While TBI influences meningeal transcriptomic alterations, Aged TBI presents a progressively active interaction network between genes associated with inflammation and potential neurodegenerative deficits.

### 3.6. Neurodegenerative disease transcriptomics are chronically impacted by aging and TBI

**Figure 6:**
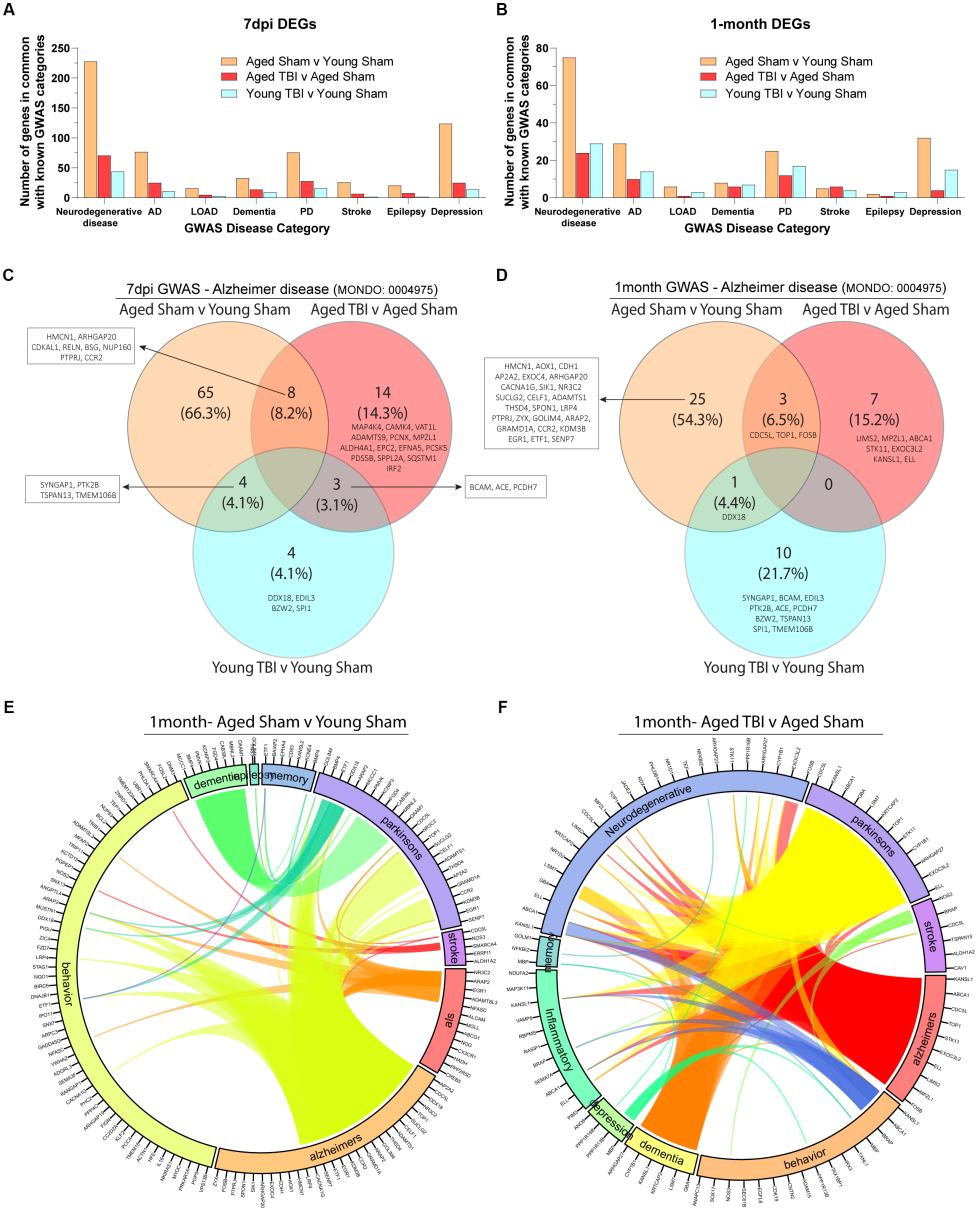
Neurodegenerative disease transcriptomics are chronically impacted by aging and TBI. **(A)** Bar graph showing the number of differentially expressed genes at 7 days post-injury that overlap with known disease categories observed in genome wide association (GWAS) studies. **(B)** Bar graph showing the number of differentially expressed genes at 1 month post injury that overlap with known disease categories observed in GWAS studies. **(C,D)** Venn diagram demonstrating the number of genes at 7 days and 1 month post injury that have been identified in Alzheimer’s disease GWAS studies. **(E,F)** Circos plot depicting how overlapping differentially expressed genes in aged and TBI mice at 1 month post injury, are associated with neurodegenerative disease states as identified in GWAS studies. The proportion of the circle’s circumference allocated to each disease represents the number of genes associated with that disease that are also differentially expressed in both aged and TBI mice. The lines connecting genes within the circle indicate which genes were shared among disease signatures.

As the effects of aging and TBI are known to increase susceptibility to other neurodegenerative disorders, we compared meningeal signatures to the human pathophysiology of known chronic neurobiological diseases at 7 days and 1 month post injury (Figures 6A,B; Supplementary Table S6). With Alzheimer’s disease as a common risk factor in TBI, we further extrapolated genomic profiles at subacute and chronic time points with age. We found that aged mice present unique profiles (66.3% at 7dpi and 54.3% at 1 month) which indicate high activity of Alzheimer’s related genes (Figures 6C,D). Transcriptomic expression related to Alzheimer’s with TBI also show differences with age at the subacute (4.1% in Young TBI and 14.3% in Aged TBI) and chronic (21.7% in Young TBI and 15.2% in Aged TBI) time points (Figures 6C,D; Supplementary Table S6). At 7 days post injury, we found 8 common genes between Aged Sham and Aged TBI, 4 common genes between Aged Sham and Young TBI, and 3 common genes between Aged TBI and Young TBI (Figure 6C). At 1 month post injury, we found 3 common genes between Aged Sham and Aged TBI, 1 common gene between Aged Sham and Young TBI, and no similar genes between Aged TBI and Young TBI (Figure 6D). Overall, Aged Sham, Aged TBI, and Young TBI present unique transcriptomic expression of disease relative to each condition. The TBI signature is consistent with other findings of upregulated *CACNA1G* with brain swelling in response to head injury. *COL25A* appears to indicate membrane-associated brain-specific collagen repair, which is unsurprising given the active extracellular matrix affected by injury. Extracellular matrix repair patterns appear to present an increase of collagens and laminins at 7 days post-injury and a dampened response 1 month of injury independent of age. Further, *ELL* appears to have a role in facilitating RNA Polymerase II elongation within epidermal proliferation, likely a result of the transcriptional regulation necessary within the extracellular matrix after injury. To determine how neurodegenerative disease is affected by TBI in the aged brain, we visualized the interactivity of disease genomics at 1 month post injury (Figures 6E,F; Supplementary Table S6). We identified that aging has significant overlap in a number of diseases including Alzheimer’s, ALS, stroke, Parkinson’s, dementia, epilepsy, and depression (Figure 6E). At the chronic time point in Young TBI 1 month post injury, several prominent genes like *CACNA1G, SENP7, ETF1, EGR1, KDM3B, CCR2, GRAMD1A, ARAP2, GOLIM4, THSD4, ADAMTS1, CELF1, SUCLG2, TOP1, NR3C2, DDX18, CDC5L,* and *AP2A2* that are related to Alzheimer’s are also linked to Parkinson’s, epilepsy, and behavior (Figure 6E). Interestingly, GWAS associations in Aged TBI 1 month post injury reveals a significantly altered genomic network pattern, where Alzheimer’s related genes *FOS8, MPZL1, LIMS2, ELL, EXOC3L2, STK11, TOP1, CDC5L, ABCA1,* and *KANSL1* are linked to neurodegeneration, inflammation, Parkinson’s, stroke and dementia. Dementia-related genes *ARHGAP27, CYP1B1, KANSL1, KRTCAP2, LSM7,* and *GBA* are also linked to altered behavior, neurodegeneration, inflammation, and Parkinson’s (Figure 6F). Notably, TBI appears to increase neurodegenerative disease interactions chronically (Figures 6E,F), which may indicate an amplified disease-state environment with aging. The neurodegenerative interaction patterns in Aged TBI (Figure 6F) are more sporadic and robust compared to Aged Sham (Figure 6E). While aging alone presents genomic associations with neurodegeneration, TBI-induced chronic inflammation in the aged brain presents further exacerbation of neurodegenerative consequences.

## 4. Discussion

In this study, we assessed the sub-acute and chronic transcriptional response in the meninges after TBI, and investigated how this response may be impacted by aging. We report that the age alone is a significant factor in driving gene expression alterations in the meningeal tissue. Specifically, aging influenced the adaptive immune response, upregulating genes involved in immunoglobulin production and the activation/regulation of B lymphocytes. In the context of TBI, both young and aged mice display sub-acute signatures of extracellular matrix remodeling, alongside upregulations in regulators related to T lymphocyte responses at 7 days post injury. This inflammatory signature is sustained at chronic timepoints, with B lymphocyte and interferon-based signatures observed at 1 month post TBI, a response that is unique to Aged TBI mice. In addition, we show that the meningeal transcriptome contains multiple inflammatory biomarker signatures and overlapping genes with those known to be associated with neurodegenerative disorders identified by Genome Wide Association Studies (GWAS).

Many studies highlight changes at baseline in the aged brain and suggest the potential for a “priming” effect that may exacerbate pathological responses, including inflammation (Perry and Holmes, 2014; Finger et al., 2022). In this study we find prominent age-related changes in meningeal immune environment. In the uninjured context, aged mice display upregulations in immunoglobulin (antibody) production, lymphocyte differentiation and B cell activation in the meninges, compared to young counterparts. This heightened adaptive immune response is in line with previous transcriptional reports showing age affects multiple lymphocyte mediated pathways under homeostatic conditions (Neutzner et al., 2023), including those important for antibody production by B cells (Bolte et al., 2023). Indeed, Brioschi et al. recently reported a lymphopoietic niche in the meninges, such that calvaria derived B cells migrate to the meninges via skull vascular channels in young mice, whereas age-associated B cells infiltrate the meninges from the periphery over time (Brioschi et al., 2021). Although further investigation is required, a combination of age-induced changes in blood brain barrier permeability and sustained barrier antigens, may explain the aged-induced increase in meningeal B cell transcripts observed in our uninjured aged mice.

Similar to studies by Bolte et al. and Neutzner et al. which contain an uninjured context, uninjured sham mice in our study are naïve without craniotomy. We chose this approach as it has been shown that the skull and vertebral bodies contain specialized bone marrow pockets that connect dural sinuses and meningeal veins, serving as niches for immune cell recruitment in response to injury (Herisson et al., 2018; Cugurra et al., 2021; Rindone et al., 2021; Bolte et al., 2023). Indeed, thinning or pressure on the skull bone can compresses the underlying meningeal space, leading to vascular damage, recruitment of neutrophils, meningeal cell death, reactive oxygen species (ROS) generation, and breaches in the glial limitans, which perpetuate inflammatory responses (Liu et al., 2011; Roth et al., 2014; Russo et al., 2018; Mason et al., 2021; Buckley and McGavern, 2022). Given the heterogeneity of TBI these barriers are usually damaged as a result of primary injury. Therefore, to investigate the spectrum of transcriptomic meningeal derived immune responses, we consider the appropriate “sham” control for this study as being a mouse that does not receive a craniotomy, since we are interested in the holistic difference between injured (which we consider encompassing mechanical injury of skull and CNS barriers as being part of the TBI as a whole), and normal brain (no craniotomy injury and no impact).

The meningeal issue in aged mice also showed baseline elevations in NF-kB, IL6, iNOS and interferon canonical pathways, a similar profile to that previously described in aged microglia (O'Neil et al., 2018; Witcher et al., 2021). Of significance, pro-inflammatory signals coming from the meninges have recently been shown to induces phenotypic changes in microglia (James et al., 2020; Da Mesquita et al., 2021; das Neves et al., 2021; van Olst et al., 2021), suggesting elevated immune signatures in the meninges influencing brain-resident immune responses may be how the aged brain is vulnerable to neurodegenerative disease processes.

Under homeostatic conditions, the brain contains a drainage system through meningeal lymphatic vasculature, which is morphologically supported by extracellular matrix (ECM) for unidirectional flow, CSF outflow, and arachnoid granulations through venus sinuses. This vasculature is characterized by the network of basal lymphatics, transporting macromolecules in protective barriers along the brain (das Neves et al., 2021). Molecular clearance may be disturbed by evidence of dysfunctional meningeal lymphatics as a result of trauma, such as peripheral vasculature entry through blood brain barrier leakage. As blood brain barrier dysfunction occurs after TBI (Cash and Theus, 2020), alterations in immune cell trafficking to and from the CNS meninges to the brain parenchyma occurs, however there is little understanding of how this immune response is directly affected by aging and severe injury. Our data provides evidence that the meningeal transcriptome is grossly affected by the functional consequences of both TBI and aging, with varying signatures at both subacute and chronic time points.

In the context of TBI, robust and complex interactions between central and peripheral cellular components occur in a time-dependent manner, and can be modified by a variety of factors, including age. Several studies have broadly outlined the cellular dynamics of this TBI-induced inflammatory response (Simon et al., 2017). Following primary injury which may cause vascular rupture and blood–brain barrier damage (Main et al., 2018), peak neutrophil recruitment occurs within the first 24–48 h (Clark et al., 1994), followed by sustained cytokine and chemokine recruitment of lymphocytes and monocyte-derived macrophages (Gyoneva and Ransohoff, 2015; Simon et al., 2017). In this process, the meninges are a key immune reservoir that may mediate this temporal peripheral response, with the meningeal tissue playing host to macrophages, dendritic cells, mast cells, neutrophils, and B and T lymphocyte (CD4+, CD8+, γδ, and Treg) subpopulations (O'Brien et al., 2017; Ribeiro et al., 2019; Neutzner et al., 2023). Although recent work has uncovered the role of meningeal driven immunity in various chronic neurodegenerative disorders (Filiano et al., 2016; Louveau et al., 2018b; Kierdorf et al., 2019; Alves de Lima et al., 2020; Brioschi et al., 2021), less is known about their role in response to moderate/severe brain injury. In TBI, it is important to recognize that during the primary injury phase, vessels suspended in the meninges are particularly vulnerable to the mechanical strain of TBI. Following the initial impact and meningeal bleeding, a series of clotting and inflammatory responses are initiated (Norris and Kipnis, 2019), often alongside fibrotic scarring and extracellular matrix repair. In our study, we find changes in gene expression related wound healing and extracellular matrix remodeling, as well as collagen fibral organization in both Young and Aged mice at 7 days post TBI. It is most likely that this response is a direct result of resolving the primary lesion with an inner fibrotic scar at the core of the injury, which seals the damage site in the days and weeks following the injury (Fernandez-Klett and Priller, 2014; Dorrier et al., 2022). When looking at top genes that may be influencing this extracellular matrix organization, multiple collagen related genes are upregulated in both TBI groups.

Alongside fibrotic scar formation, surveilling T cells can recognize danger-associated molecular patterns (DAMPs) released by necrotic cells in the brain into the CSF as a result of the initial trauma (Gadani et al., 2015). The exact function of these T cells in TBI is still unclear, with evidence suggesting that brain-penetrating T cells may limit neuronal damage after mechanical injury (Ellwardt et al., 2016), while regulatory T cell depletion may induce reactive astrogliosis and worsen outcomes after TBI (Kramer et al., 2019). To this end, we found that the meningeal tissue in Young and Aged TBI mice share conserved upstream regulators of T cell responses, specifically T-helper-17 (Th17) lineage, at 7 days post injury. This is of interest as Th17 cells represent a distinct population which differentiates and promote inflammation in presence of IL-6 and transforming growth factor-β (TGFβ; Cipollini et al., 2019; Alam et al., 2020). Indeed, here we show IL-6 is an upstream regulator in Young TBI mice, while TGFβ is activated in both Young and Aged mice. While TGFβ may have a role in T cell differentiation, studies also evidence its ability to activate retinoic acid signaling pathways in meningeal fibroblasts, stimulate arachnoid barrier cells and facilitate reconstruction of the blood-CSF barrier (Cha et al., 2014).

In line with previous study by Bolte et al. (2023) utilizing a closed mild head impact model, we find that aging alone increases meningeal immunoglobulin production, immune system processes, lymphocyte activation, and B cell activation, representing heightened adaptive immune responses. This baseline enhanced immune status may contribute to sustained chronic inflammation, as Aged TBI mice displayed upregulated antibody based immune responses at 1 month post injury. Interestingly, B cells have recently been shown to have immunoregulatory effects on meningeal myeloid cells, including the regulation of IFN-γ signaling in the meninges (Lynall et al., 2021), thus suggesting the potential for this sustained meningeal response in Aged TBI mice to be mediated by splenic B cells and INF-γ-mediated signaling.

At 1 month post injury, upstream regulators further implicated inflammatory mediators in both young and aged mice. Young TBI appears to presents an increase of SORL1 activation and decrease in chemokine activation (IL1B, IKBKG). In Aged TBI mice at 1 month post injury, unique CD3 and IL33 upstream signatures are observed, alongside interferon-based signatures compared to Young TBI mice. In fact, interferon regulation is dampened sub-acutely, but dramatically increased at 1 month post injury in the aged brain, which suggests an accumulated adaptive immune response that appears to surpass the non-injured baseline. In contrast, interferon regulation remains relatively consistent at all time points in young TBI, and has more activity in the non-injured young paradigm, which indicates a significant boost of interferon mediators 1 month post-injury. Indeed, this amplified interferon-based response has been seen in the resident immune cells of the brain in aged animals after TBI (Barrett et al., 2021; Wangler et al., 2022), however our data suggest that future studies into targeting meningeal specific interferon responses may be warranted.

While it is known that TBI is a risk-factor for neurodegenerative diseases (Wu et al., 2021; Migliore and Coppede, 2022), the question of why many patients become vulnerable to other neurological disorders is still unclear. Indeed, geriatric head injury yields poor clinical outcomes with proposed mechanisms associated with inflammatory cascades (Benko et al., 2019). Therefore, to provide insight into this question and infer translatability to human pathophysiology, we analyzed the intersection of the molecular signals from our meningeal dataset with transcripts from human genome-wide association studies (GWAS), which identify known traits and diseases (Cloney, 2016; Manzoni et al., 2018; Skol et al., 2020; Zhou et al., 2021; Jia et al., 2022; Rinki, 2022). The importance of this comparison of overlapping genes identified within our dataset and GWAS, is to highlight the biological relevance for the role of the identified genes and illustrate the potential of their mechanistic role within the meninges after TBI. We show common transcriptomic markers in aging and TBI overlap in a number of traits, including inflammatory biomarkers, memory and cognition. We also show an intersecting epigenetic network context to these age- and injury-related mechanisms, and show the meningeal architecture contains signature associations with neurodegenerative disease, such as dementia, Alzheimer’s, ALS, stroke, Parkinson’s, epilepsy and depression. Interestingly, Alzheimer disease is a prominent GWAS disease associated with TBI, and is known to accelerate pathogenically with mild microvascular injury (Wu et al., 2021). While we demonstrate unique Alzheimer’s disease genomic associations with age and injury in the meningeal compartment containing vascular and lymphatic structures, further investigation is necessary to understand how these molecular effects are functionally mediated. Overall, these integrations provide important insights into meningeal specific candidates, and list potential targets for intervention of transcripts that play key roles in neurodegenerative disease processes.

In this study, we focused on the bulk transcriptional signature that occurs in meninges after TBI and assessed the influence of age on this response. This approach provided valuable holistic insights into all responses from extracellular matrix repair to inflammatory cascades and transcripts that can identify cell specific responses. However, a limitation of this study is that it was only conduced in male mice and not at the single cell level. Indeed, a full repertoire of immune cells including macrophages, B cells, T cells, NK cells, dendritic cells, plasmacytoid dendritic cells, and neutrophils have been previously identified in the meninges in the context of TBI (Bolte et al., 2023), and mapping inflammatory transcripts to a particular cell type in this scenario was not possible. In future studies, it will be important to further interrogate the functions of all cell populations in TBI at a level of appropriate resolution both male and female mice. In addition, these cells may be localized to various layers of the complex meningeal architecture (dura, pia mater, arachnoid) as meningeal immune cells have been shown to have a distinct sub-meningeal distribution (Rua and McGavern, 2018; Alves de Lima et al., 2020).

While our data set provides insight into potential changes to the meninges in the TBI and aging, it remains unclear specifically as to which cell type is affected, and at which distinct meningeal layer.

## 5. Conclusion

Our data demonstrates the time-course of meningeal specific signatures and provides insights into how age and injury interact, leading to chronic neuroinflammation. We show that at baseline, age alters the meningeal transcriptome with increased expression of signatures related to antibody production and B cell responses. Following TBI, this adaptive immune profile is sustained chronically in the aged meninges, alongside activation pro-inflammatory regulators including the interferons. Understanding the meningeal contribution to neuroinflammation, and how this response is altered by age, will provide us with valuable knowledge to identify mechanisms by which age renders the brain vulnerable to worse outcomes in TBI and neurodegenerative disorders.

## Data availability statement

The datasets presented in this study can be found in online repositories. The names of the repository/repositories and accession number(s) can be found in the article/Supplementary material.

## Ethics statement

All procedures were conducted in accordance with protocols approved by the Georgetown University Animal Care and Use Committee.

## Author contributions

RB, AH, and BM conducted TBI surgeries and molecular experiments. BM and MB designed and funded the experiments. RB and BM analyzed the data, interpreted the results, and wrote the manuscript. RB, AH, MB, and BM contributed to editing the manuscript. All authors contributed to the article and approved the submitted version.

## Funding

This work was supported by the National Institutes of Health (NIH)/National Institute of Neurological Disorders and Stroke (NINDS; R01NS107370 to MB) and the Georgetown Office of Advancement, Georgetown University Research Grant awarded to BM.

## Conflict of interest

The authors declare that the research was conducted in the absence of any commercial or financial relationships that could be construed as a potential conflict of interest.

## Publisher’s note

All claims expressed in this article are solely those of the authors and do not necessarily represent those of their affiliated organizations, or those of the publisher, the editors and the reviewers. Any product that may be evaluated in this article, or claim that may be made by its manufacturer, is not guaranteed or endorsed by the publisher.

## Footnotes

^1^https://software.broadinstitute.org/morpheus

^2^http://geneontology.org/

^3^https://www.ebi.ac.uk/gwas
