# Supplementary material for: Traumatic brain injury induces an adaptive immune response in the meningeal transcriptome that is amplified by aging
Source: Front Neurosci. 2023 Jul 31;17:1210175. doi: 10.3389/fnins.2023.1210175 (PMC10425597; doi:10.3389/fnins.2023.1210175)
Supplement: Supplementary file 7 [file Table_7.DOCX]

**Supplementary Methods Table 1. TaqMan gene expression assays and methods used in RT- QPCR**

RT-QPCR validation was performed using RNA from the same samples that were processed through RNA-seq, where concentration was first determined using a NanoDrop 100 spectrophotometer (Thermo Scientific) and reverse transcribed (1 ug/RNA) into complementary DNA (cDNA) with a High-Capacity RNA-to-DNA Reverse Transcription Kit (4368814, Applied Biosystems, Foster City, CA) according to manufacturing protocols. RT-QPCR was completed using standard 384-well plates (4309849, Applied Biosystems) with TaqMan Fast Advanced Master Mix (4444556, Thermo Fisher) and Taqman probes Igkv4-53, C1qtnf6, Ighg1, and Pcp2, under the cycle conditions of 50 °C for 2 minutes, 95 °C for 20 seconds, (95 °C for 1 second, 60 °C for 20 seconds) with 40 cycles. Applied Biosystems SDS 2.4 software was used to indicate threshold cycle values (Ct), where fold change (FC) was calculated under (2^−ΔΔCt^) method.

| Gene Name | Assay ID |
| --- | --- |
| C1qtnf6 | Mm00511605_m1 |
| Col1a1 | Mm00801666_g1 |
| Ighg1 | Mm05833521_m1 |
| Igkv4-53 | Mm01611505_g1 |
| Pcp2 | Mm00435514_m1 |
