# Supplementary figures and images for: Traumatic brain injury induces an adaptive immune response in the meningeal transcriptome that is amplified by aging
Source: Front Neurosci. 2023 Jul 31;17:1210175. doi: 10.3389/fnins.2023.1210175 (PMC10425597; doi:10.3389/fnins.2023.1210175)

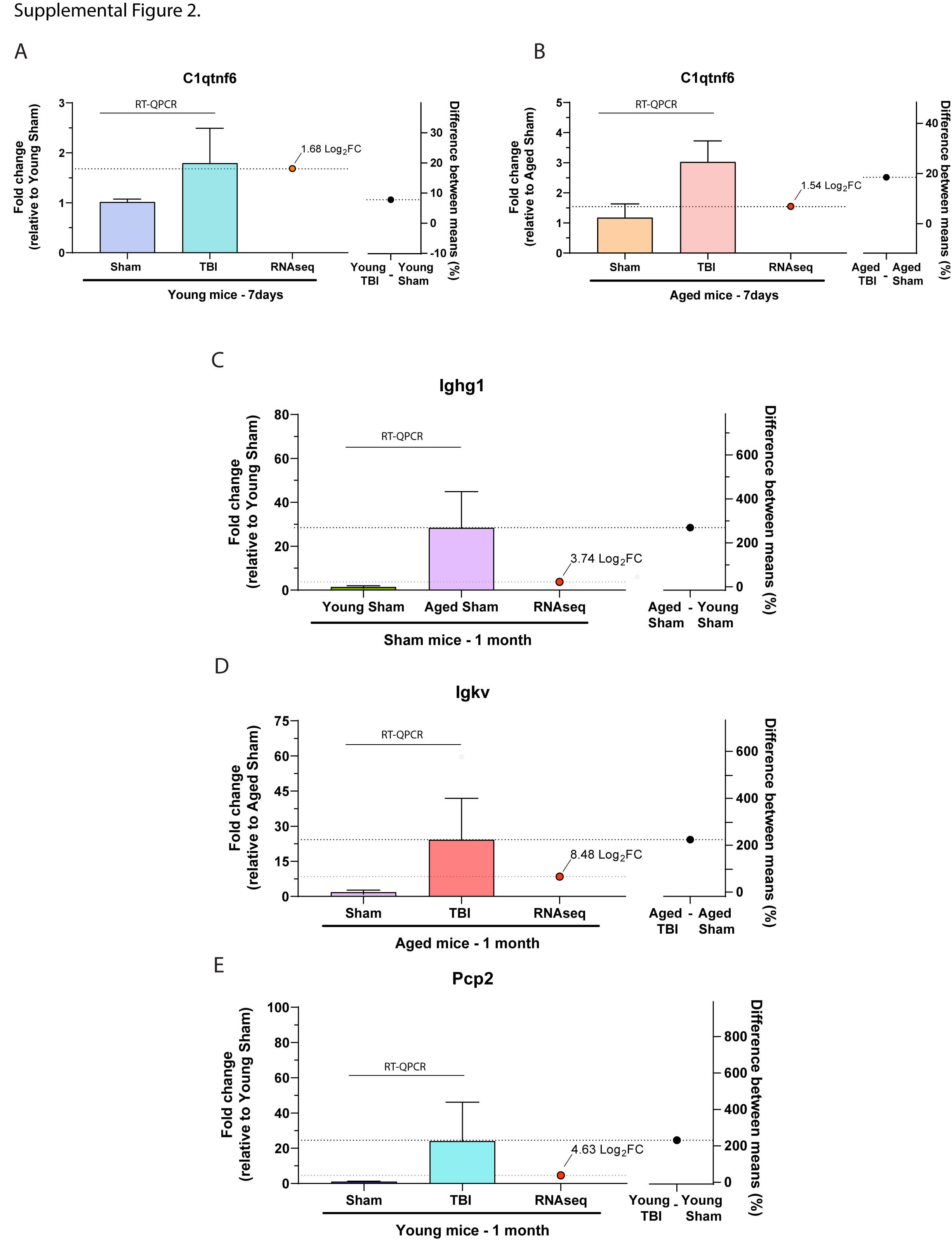

Supplement: Supplementary file 10 [file Image_1.TIF]

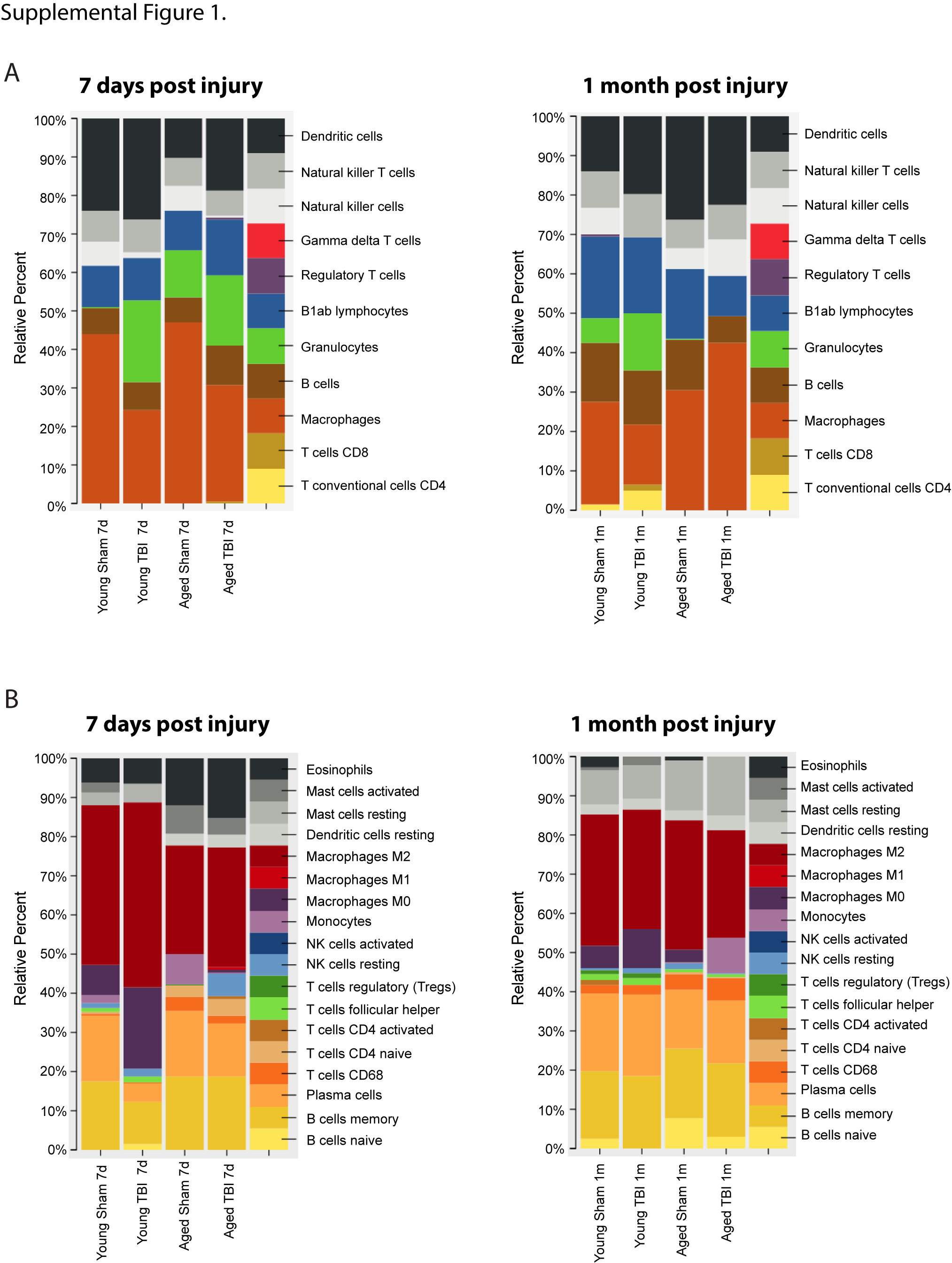

Supplement: Supplementary file 11 [file Image_2.TIF]
